# Transcriptomics Reveal Several Gene Expression Patterns in the Piezophile *Desulfovibrio hydrothermalis* in Response to Hydrostatic Pressure

**DOI:** 10.1371/journal.pone.0106831

**Published:** 2014-09-12

**Authors:** Amira Amrani, Aurélie Bergon, Hélène Holota, Christian Tamburini, Marc Garel, Bernard Ollivier, Jean Imbert, Alain Dolla, Nathalie Pradel

**Affiliations:** 1 Aix-Marseille Université, Université du Sud Toulon-Var, CNRS/INSU, IRD, MIO, UM110, Marseille, France; 2 Inserm, U1090, TGML/TAGC, Marseille, France; 3 Aix Marseille Université, UMR_S 1090, TGML/TAGC, Marseille, France; 4 Aix-Marseille Université, CNRS, LCB-UMR7283, Marseille, France; Oak Ridge National Laboratory, United States of America

## Abstract

RNA-seq was used to study the response of *Desulfovibrio hydrothermalis*, isolated from a deep-sea hydrothermal chimney on the East-Pacific Rise at a depth of 2,600 m, to various hydrostatic pressure growth conditions. The transcriptomic datasets obtained after growth at 26, 10 and 0.1 MPa identified only 65 differentially expressed genes that were distributed among four main categories: aromatic amino acid and glutamate metabolisms, energy metabolism, signal transduction, and unknown function. The gene expression patterns suggest that *D. hydrothermalis* uses at least three different adaptation mechanisms, according to a hydrostatic pressure threshold (HP_t_) that was estimated to be above 10 MPa. Both glutamate and energy metabolism were found to play crucial roles in these mechanisms. Quantitation of the glutamate levels in cells revealed its accumulation at high hydrostatic pressure, suggesting its role as a piezolyte. ATP measurements showed that the energy metabolism of this bacterium is optimized for deep-sea life conditions. This study provides new insights into the molecular mechanisms linked to hydrostatic pressure adaptation in sulfate-reducing bacteria.

## Introduction

Marine ecosystems represent the major volume of the biosphere and occupy the largest surface of the planet. Approximately 90% of this volume is at depths below 1000 m [1]. One characteristic of this deep-sea environment is the high hydrostatic pressure encountered by the indigenous organisms, which are thus called “piezophiles” [2]. In this environment, sulfate-reducing bacteria (SRB) play a key role in the coupling of the carbon and sulfur biogeochemical cycles by utilizing sulfate as the terminal electron acceptor for the oxidation of organic matter [3]–[5]. However, although they are widely distributed across the Earth, few SRB strains, and only two *Desulfovibrio* spp., have been isolated from the deep-sea biotope. *Desulfovibrio piezophilus* C1TLV30 was isolated from wood falls at a depth of 1,700 m in the Mediterranean Sea [6], and *Desulfovibrio hydrothermalis* AM13 was isolated from a deep-sea hydrothermal vent in the East-Pacific Rise, at a depth of 2,600 m [7], [8]. This latter bacterium grows preferentially at hydrostatic pressure that is 260 times higher (26 MPa) than atmospheric pressure (0.1 MPa), corresponding to the existing *in situ* pressure at the site of isolation [7]. Therefore, this bacterium must have evolved particular adaptive mechanisms to address various hydrostatic pressure conditions.

Recent works on other deep-sea organisms, such as *Photobacterium profundum SS9*, have revealed that regulation at the transcriptome level plays an important role in hydrostatic pressure adaptation [9]–[11]. Moreover, studies performed on *D. piezophilus* C1TLV30 have suggested that pressure affects several cellular functions, particularly amino acid transport and metabolism and sulfate-reducing activity [12]. However, transcriptome-level studies of adaptations to diverse pressure conditions and details of the downstream target network have not been reported for SRB. In this study, we used RNA-seq to interrogate variations in gene expression with hydrostatic pressure in *Desulfovibrio hydrothermalis* AM13. This technique allows the entire transcriptome to be surveyed in a high-throughput, sensitive, and quantitative manner. Whole-genome expression patterns of cells cultured at different hydrostatic pressures were thus determined and compared by sequencing cDNAs using a next-generation sequencing method. The transcriptomes were examined in cells cultured at three different hydrostatic pressures: the *in situ* pressure from which *D. hydrothermalis* has been isolated (26 MPa); an intermediate pressure (10 MPa) corresponding to a depth of 1,000 m, which has been reported to be the critical depth for the deep-sea environment [13]; and atmospheric pressure (0.1 MPa). A comparison of these conditions highlights specific genes and metabolic pathways that are involved in the adaptation of this bacterium to hydrostatic pressure and suggests the existence of several adaptive mechanisms that are engaged at different hydrostatic pressure levels.

## Materials and Methods

### Desulfovibrio hydrothermalis AM13 growth conditions

*Desulfovibrio hydrothermalis* AM13 was grown anaerobically at the optimal temperature of 30°C, at 0.1 MPa (atmospheric pressure), 26 MPa or 10 MPa, for 40 h in 20 ml of the medium described by Alazard *et al.* [7]. These cultures were used to inoculate fresh medium (100 ml) for RNA preparation and biochemical experiments at each corresponding hydrostatic pressure. These large-scale cultures were inoculated at a 1∶20 ratio, and the cells were grown until the late exponential growth phase (OD_600_ = 0.6). All cultures were carried out in duplicate. Cells were treated as indicated below for either RNA preparation or intracellular metabolite extraction.

### RNA isolation

For total RNA isolation, cells from each hydrostatic pressure condition were harvested by centrifugation at 6000× *g* for 20 min at 4°C and washed once with 20 ml of 0.1 M Tris-HCl 0.15 M NaCl buffer (pH 7.6). The pellets were rinsed three times with 10 mM Tris-HCl (pH 8.0) RNAse-free buffer and finally resuspended in 200 µl of 10 mM Tris-HCl, 1 mM EDTA (pH 8.0) RNase-free buffer. Total RNA was isolated using the High Pure RNA Isolation Kit (Roche Diagnostics) according to the manufacturer's instructions, with an extra DNase I digestion step to eliminate contaminating DNA. RNA quality was assessed on an Agilent 2100 Bioanalyzer (Agilent Technologies, Santa Clara, CA, USA). RNA was quantified by spectrophotometry at 260 nm (Nanodrop 2000c Thermo Scientific). RNA was prepared from two independent cultures for each pressure condition (two RNA pools for each condition) and was used further for RNA-seq experiments.

### Enrichment of mRNA from total RNA

For mRNA enrichment, 23S and 16S rRNA were depleted using a Ribo-Zero Magnetic Kit (Gram-Negative Bacteria by Epicentre, Illumina, Cat. No. MRZGN126) according to the manufacturer's instructions. Briefly, 5 µg of total RNA from each sample was treated with Ribo-Zero rRNA Removal Solution. rRNA was then removed using magnetic beads from the Ribo-Zero Magnetic Core Kit (Cat. No. MRZ116C). The ribo-depleted samples were purified by ethanol precipitation and eluted in 13 µl of RNase-free water. The concentration of the resulting RNA was determined using a NanoDrop ND-1000 Spectrophotometer (NanoDrop Technologies, Wilmington, DE, USA); 8–16% of the input RNA was recovered after purification. RNA integrity was assessed on the Agilent 2100 Bioanalyzer with the RNA 6000 Pico chip Kit.

### cDNA library construction for RNA-seq

Strand-specific library construction was carried out using the SOLiD Total RNA-seq Kit (Life Technologies). Approximately 0.35 µg of ribo-depleted RNA was subjected to enzymatic mRNA fragmentation using RNase III from the SOLiD Total RNA-seq Kit. The fragmented RNA was then concentrated using the Invitrogen RiboMinus Concentration Module according to the manufacturer's protocol. The quantity and quality of the resulting RNA were assessed using a Qubit Fluorometer (Life Technologies) and an Agilent 2100 Bioanalyzer with the RNA 6000 Pico chip Kit.

Fragmented RNA (0.1 µg) was subjected to cDNA synthesis. After hybridization and ligation of the SOLiD Adaptor, RNA was reverse-transcribed at 42°C for 30 min. The resulting cDNA was purified twice using the Agencourt AMPure XP Reagent. The eluted cDNA was then PCR amplified (95°C for 5 min; 10 cycles of 95°C for 30 sec, 62°C for 30 sec, and 72°C for 30 sec; 72°C for 7 min) using a SOLiD 5′ primer and a SOLiD 3′ primer specific for each sample (barcoding allowed us to pool the samples for sequencing). The resulting product was then purified using the same technique used for cDNA purification (Agencourt AMPure XP). The quality and integrity of the amplified DNA was confirmed using a NanoDrop ND-1000 spectrophotometer and an Agilent 2100 Bioanalyzer with DNA 1000 Kit. The cDNA libraries were constructed from two independent experiments for each pressure growth condition.

### Sequencing

The libraries were amplified with the SOLiD EZ Bead Amplifier. The samples had previously been prepared using the SOLiD EZ Bead Emulsifier, which allows the mixing of the aqueous phase containing the SOLiD EZ Bead, library template, and primers with the oil phase (Oil Master Mix). The preparation was then amplified by PCR (amplifier software scale E80). After the run was complete, the samples were purified using the SOLiD EZ Bead Enricher according to manufacturer's instructions. The P2-enriched beads were then concentrated, and the 3′ends were modified as described in the manufacturer's protocol. Paired-end stranded RNA-seq with ribo-depleted total RNA was performed using a 5500 xl SOLiD high-throughput sequencer (Life Technologies). The RNA-seq data have been deposited in the NCBI Gene Expression Omnibus (GEO, http://www.ncbi.nlm.nih.gov/geo/) under accession number GSE55745. Accession number in the NIH Short Read Archive is SRP039611.

### Bioinformatic data analysis

The obtained reads were mapped to the *D. hydrothermalis* AM13 genome sequences (MicroScope accession number: DESAMv2_DESAMv2) using the Life Technologies Bioscope Genomic Analysis Software. To estimate the level of transcription for each gene, the number of reads that mapped within each annotated coding sequence (CDS) was determined using the multiBamCov program from the BEDTools suite [14]. For each pressure condition, the data obtained from the two independent cDNA libraries (replicates 1 and 2) were added. To enable comparison of the expression levels between both different RNA-seq experiments and different genes within the same experiment, it was necessary to normalize the read counts. The R package DESeq was used to analyze the differential expression of genes between the different culture conditions [15]. Read counts for the different conditions of culture were compared to determine the log2 fold change in the abundance of each transcript. P-values were calculated and adjusted for multiple testing using the false discovery rate controlling procedure [16], [17]. An adjusted P-value<0.1 was considered statistically significant.

### Metabolite extraction

For intracellular glutamate quantitation, cultures were performed as described above for RNA preparation. Cells were immediately harvested from 2 ml cultures by centrifugation at 15,000 × *g* for 3 min and then washed with 1 ml of 200 mM NaCl solution. The pellet was resuspended with 0.5 ml boiled water and incubated for 15 min at 100°C, followed by incubation on ice for 10 min. The extract was centrifuged at 15,000 × *g* for 5 min to eliminate cell debris, and the supernatant was snap-frozen and stored at −80°C. Intracellular glutamate was measured using the fluorometric Glutamate Assay kit (Abcam, UK). Samples were plated in 96-well black microplates (Costar 96-well Assay Plates, Black Polystyrene) for fluorescence readings on a TECAN Infinite M200 plate reader, and data were obtained using the “i-control 1.6” software.

The same cultures were used for ATP and ADP quantitation. The ATP measurements were performed using the ATP Biomass Kit HS (BioThema, Sweden) according to manufacturer's instructions. Samples (20 µl of culture) were plated in 96-well microplates (Nunc F96 MicroWell White Polystyrene Plate, Thermo Fisher Scientific) with 20 µl of Extractant B/S and 160 µl of reconstituted ATP Reagent HS. Assays were calibrated by adding 10 µl of 100 nmol/l internal ATP standard. The ADP/ATP ratio was determined using the bioluminescent ADP/ATP Ratio Assay kit (Abcam, UK) according to manufacturer's instructions. Samples were prepared using the TCA extraction method, as described by Zhang *et al*. [18]. Briefly, 0.2 ml of culture was quickly mixed with 0.2 ml of solution containing 1% TCA and 25 mM EDTA and incubated on ice for 10 min. The extract was then centrifuged for 10 min at 15,000 × *g*. The supernatant was harvested and TCA was neutralized to pH 6 by the addition of 600 µl of 20 mM Tris-acetate, pH 7.75. Luminescence was measured using a Luminoskan Ascent plate reader (Thermo Scientific), and the data were treated using the Ascent software v.2.6 (Thermo Scientific).

## Results

### RNA-seq sequence alignment and transcript level estimation

**Figure 1:**
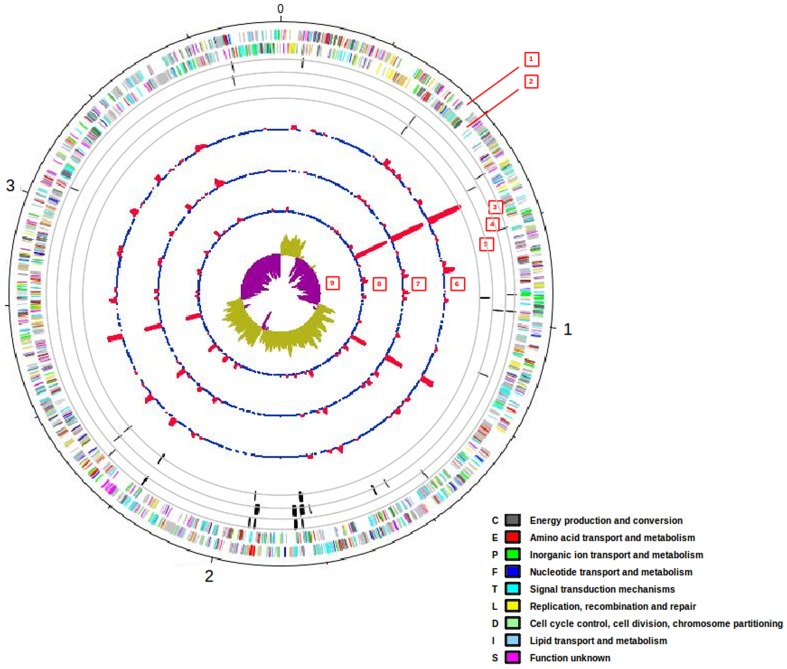
Genomic organization of *D. hydrothermalis* overlaid with differentially expressed genes and expression levels obtained from RNA-seq experiments. Moving from the outside inward, the circles represent 1, 2) CDS on the plus and minus strands of the genome; loci of differentially expressed genes in 3) 26 MPa *vs.* 0.1 MPa, 4) 10 MPa *vs.* 0.1 MPa, 5) 26 MPa *vs.* 10 MPa; coverage (from BAM format) for 6) 26 MPa, 7) 10 MPa, 8) 0.1 MPa; 9) GC skew.

To identify genes and metabolic pathways involved in the adaptation of *D. hydrothermalis* to hydrostatic pressure, gene transcription profiles from cells grown under three hydrostatic pressures, *i.e.* 26, 10 and 0.1 MPa, were analyzed using RNA-seq technology. cDNA libraries from two independent biological replicates were prepared for each pressure condition. Each library was sequenced separately [19]. Little background noise was detected between the sequenced lanes, and because of the very high correlation and the high number of reads obtained, it was determined that one lane was sufficient to sequence each sample for our analysis. After filtering out low-quality reads and primer contamination, the lowest number of properly paired reads among the 6 samples was 14.8 million. A high proportion of properly paired reads (higher than 25.6%) mapped back onto the genome and was uniformly distributed across the genome, indicating the absence of sequencing coverage bias (Figure 1, Table S1).

**Figure 2:**
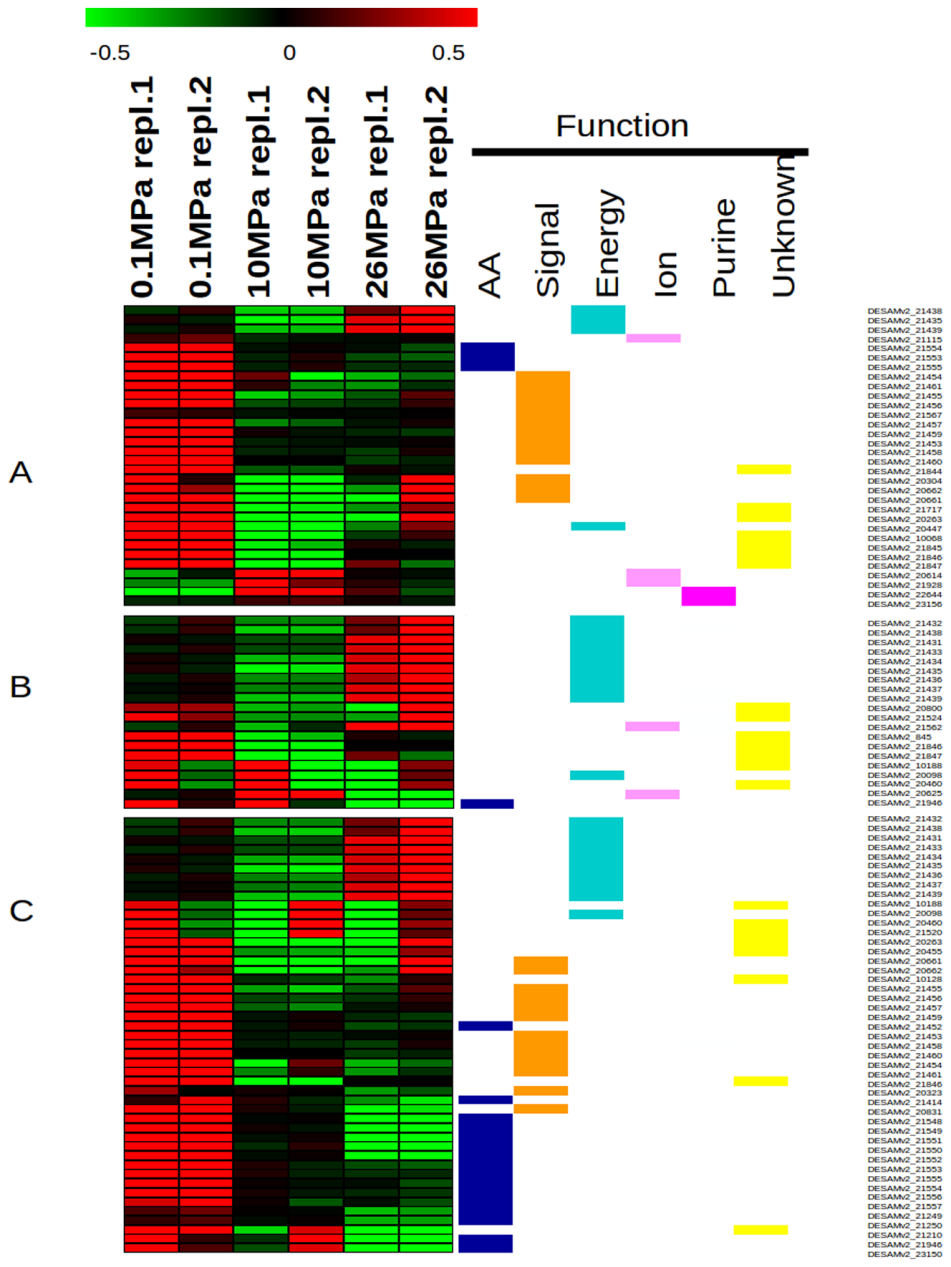
Heatmap of *D. hydrothermalis* gene expression changes with pressure. Normalized counts obtained with DESeq, transformed into log2 (RPKM+1), were used to generate a heatmap showing over-expressed (red) and under-expressed (green) genes with 2 replicates for 3 pressure conditions (0.1, 10 and 26 MPa). Three clusters corresponding to DESeq pressure-regulated genes, with an adjusted P-value<0.1, are shown (A: 10 *vs.* 0.1 MPa; B: 26 *vs.* 10 MPa; C: 26 *vs.* 0.1 MPa). Functional annotation corresponding to pressure-regulated genes is displayed.

To compare the manner in which expression patterns depended upon pressure, a heat map was constructed and hierarchical clustering was performed using the genes that were determined to be responsive to pressure conditions based on DESeq data treatment (adjusted P-value <0.1). According to their functional annotations (Figure 2), three dominant pressure-driven patterns of up-regulation (red) and down-regulation (green) emerged when comparing 10 *vs.* 0.1, 26 *vs.* 10, and 26 *vs.* 0.1 MPa. Signal transduction and regulatory functions were over-represented in the 10 *vs.* 0.1 MPa comparison, whereas energy production and conversion functions were prominent in the 26 *vs.* 10 MPa comparison. Interestingly, when comparing the *in situ* hydrostatic pressure (26 MPa) to atmospheric pressure (0.1 MPa), amino acid transport and biosynthesis functions were over-represented in addition to the two previously mentioned classes.

**Figure 3:**
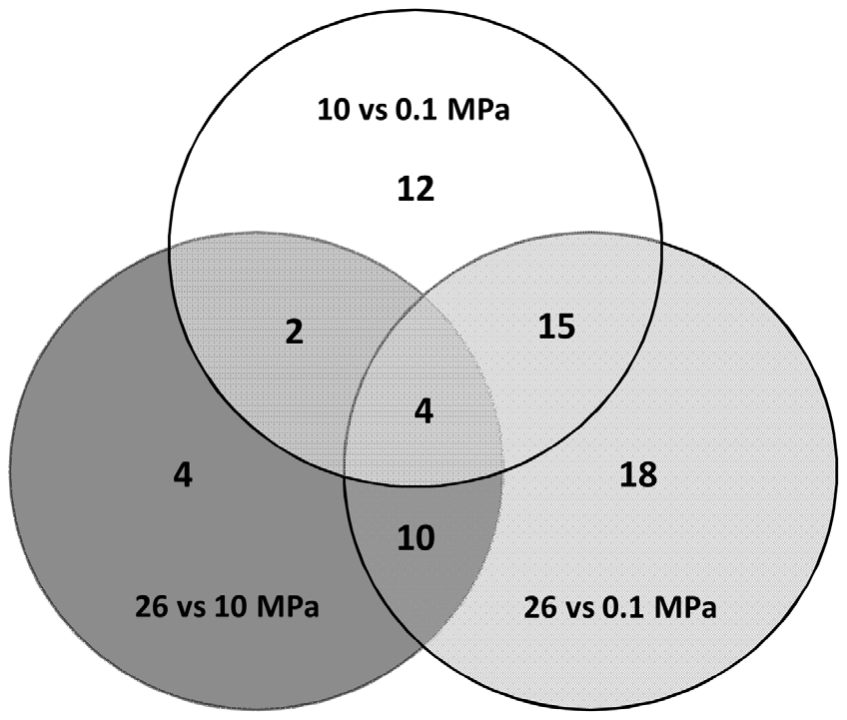
Venn diagram showing numbers of differentially expressed genes in *D. hydrothermalis* between the hydrostatic pressures of 0.1 MPa, 10 MPa and 26 MPa (adjusted P-value<0.1).

Overall, 65 genes were differentially expressed depending on the hydrostatic pressure growth conditions (Figure 3, Table 1). Among these, 33 genes were differentially expressed when cells were cultured at 10 MPa compared to 0.1 MPa, and most were down-regulated at 10 MPa (28 out of 33). When cells were cultured at 26 MPa, 47 genes were differentially expressed compared to 0.1 MPa. Again, a large majority were down-regulated (38 out of 47). On the other hand, when the transcriptomes of cells grown at 10 MPa and 26 MPa were compared, 15 of the 20 differentially expressed genes were up-regulated at 26 MPa (Table 1). These results indicate that gene expression changed more when going from atmospheric pressure (0.1 MPa) to high hydrostatic pressure (10 or 26 MPa) than between the two high hydrostatic pressure conditions (10 and 26 MPa). The overlap in the pressure-regulated expression patterns between the three conditions was quite low (Figure 3). Only 4 genes were differentially expressed in the three conditions, while the maximum overlap was found between 10 MPa *vs.* 0.1 MPa and 26 MPa *vs.* 0.1 MPa. These results suggest that *D. hydrothermalis* may engage different response mechanisms depending on a hydrostatic pressure threshold: a first-level response may occur between 0.1 and 10 MPa, with a second-level response occurring between 0.1 MPa and a hydrostatic pressure threshold above 10 MPa, hereafter referred to as HP_t_.

**Table 1:** Differentially expressed genes between the 0.1 MPa, 10 MPa, and 26 MPa growth conditions (adjusted P value<0.1).

Gene ID	Product	Log2 fold change, 10 *vs.* 0.1 MPa	Log2 fold change, 26 *vs.* 10 MPa	Log2 fold change, 26 *vs.* 0.1 MPa	COG
*Amino acid transport and biosynthesis*					
DESAMv2_21249	ATP-hydrolyzing 5-oxoprolinase			−2.917	E
DESAMv2_21250	ATP-hydrolyzing 5-oxoprolinase			−2.613	E
DESAMv2_21414::trpB	Tryptophan synthase β subunit			−3.025	E
DESAMv2_21452::glnB	Nitrogen regulatory protein P-II			−2.495	E
DESAMv2_21548	Uncharacterized aldolase aq_1554			−3.577	G
DESAMv2_21549	3-dehydroquinate synthase			−3.400	E
DESAMv2_21550::pheA	Chorismate mutase/Prephenate dehydratase			−3.600	E
DESAMv2_21551::aroA	3-phosphoshikimate 1-carboxyvinyltransferase			−2.888	E
DESAMv2_21552::tyrA	Chorismate mutase/Prephenate dehydrogenase			−3.044	E
DESAMv2_21553::trpE	Anthranilate synthase component 1	−2.703		−3.210	E
DESAMv2_21554::trpG	Glutamine amidotransferase of anthranilate synthase component 2	−2.731		−3.851	E
DESAMv2_21555::trpD	Anthranilate phosphoribosyltransferase	−2.604		−3.494	E
DESAMv2_21556::trpC	Indole-3-glycerol-phosphate synthase			−2.645	E
DESAMv2_21557::trpF	N-(5′-phosphoribosyl) anthranilate isomerase			−2.466	E
DESAMv2_21946::glnA	Type 3 glutamine synthetase		−3.076	−3.305	E
DESAMv2_23150	Extracellular ligand-binding receptor, glutamate receptor-related			−2.530	E
*Energy production and conversion*					
DESAMv2_20098::fldA	Flavodoxin		−2.717	−2.835	C
DESAMv2_20447::yiaY	Fe-containing alcohol dehydrogenase	−3.742			C
DESAMv2_21431	response regulator receiver, CheY-like		3.196	2.862	T
DESAMv2_21432	UspA domain protein		3.582	2.346	T
DESAMv2_21433::hmcF	Protein DVU_0531, HmcF		4.200	3.144	C
DESAMv2_21434::hmcE	Protein DVU_0532, HmcE		4.489	3.162	C
DESAMv2_21435::hmcD	Protein DVU_0533, Hmc operon protein 4	−3.594	4.897	3.345	C
DESAMv2_21436::hmcC	Protein DVU_0534, HmcC		4.483	2.775	C
DESAMv2_21437::hmcB	Protein DVU_0535; HmcB		4.917	3.148	C
DESAMv2_21438::hmcA	High-molecular-weight cytochrome c	−3.004	5.728	2.724	C
DESAMv2_21439	protein of unknown function	−2.922	5.669	2.746	
*Inorganic ion transport*					
DESAMv2_20613::feoB	Fe^2+^ transporter B subunit	2.361			P
DESAMv2_20614::feoA	Fe^2+^ transporter A subunit	2.732			P
DESAMv2_20625::feoA	Fe^2+^ transporter A subunit		−2.756		P
DESAMv2_21115	Fe^3+^-siderophore transporter permease	−4.248			P
DESAMv2_21562	Outer membrane efflux protein		2.780		MU
DESAMv2_21928	Sirohydrochlorin cobaltochelatase CbiKp	2.939			H
*Signal transduction and regulatory functions*					
DESAMv2_20304	Heat shock protein Hsp20	−2.462			O
DESAMv2_20323	Signal transduction histidine kinase			−3.024	T
DESAMv2_20661	Heat shock protein Hsp20	−3.041		−2.591	O
DESAMv2_20662	Heat shock protein Hsp20	−3.275		−2.525	O
DESAMv2_20831	Transcriptional regulator, MarR family			−2.581	K
DESAMv2_21453	predicted membrane protein	−3.083		−3.045	GEPR
DESAMv2_21454	predicted membrane protein	−3.465		−4.268	GEPR
DESAMv2_21455	response regulator receiver, CheY-like	−3.305		−2.850	T
DESAMv2_21456	Signal transduction histidine kinase	−4.621		−3.939	T
DESAMv2_21457	response regulator receiver, CheY-like	−4.759		−4.038	T
DESAMv2_21458	response regulator receiver, CheY-like	−4.720		−4.894	T
DESAMv2_21459	response regulator receiver, CheY-like	−4.432		−4.809	T
DESAMv2_21460	predicted membrane protein	−4.020		−4.478	R
DESAMv2_21461	conserved protein of unknown function	−3.615		−4.324	
DESAMv2_21567::fliK	Flagellar hook-length control protein	−3.553			NT
*Purine biosynthesis*					
DESAMv2_22644::glyA	Serine hydroxymethyltransferase	2.773			E
DESAMv2_23156::purU	Formyltetrahydrofolate hydrolase	3.988			F
*Unknown function*					
DESAMv2_10068	conserved protein of unknown function	−2.967			J
DESAMv2_10128	conserved protein of unknown function			−2.930	
DESAMv2_10188	conserved protein of unknown function		−3.725	−3.141	
DESAMv2_20263	conserved protein of unknown function	−2.826		−3.239	
DESAMv2_20455	protein of unknown function			−2.520	
DESAMv2_20460	conserved protein of unknown function		−2.978	−3.173	
DESAMv2_20800	conserved protein of unknown function		2.719		
DESAMv2_21210	conserved protein of unknown function			−2.358	S
DESAMv2_21520	protein of unknown function			−2.468	
DESAMv2_21524	periplasmic protein of unknown function		3.242		
DESAMv2_21717	conserved protein of unknown function	−2.543			T
DESAMv2_21844	membrane protein of unknown function	−3.799			
DESAMv2_21845	protein of unknown function	−5.764	3.755		T
DESAMv2_21846	membrane protein of unknown function	−5.715	2.666	−2.829	
DESAMv2_21847	membrane protein of unknown function	−5.798	3.573		S

Log2-fold change is indicated *vs.* the average counts over all six samples.

**Figure 4:**
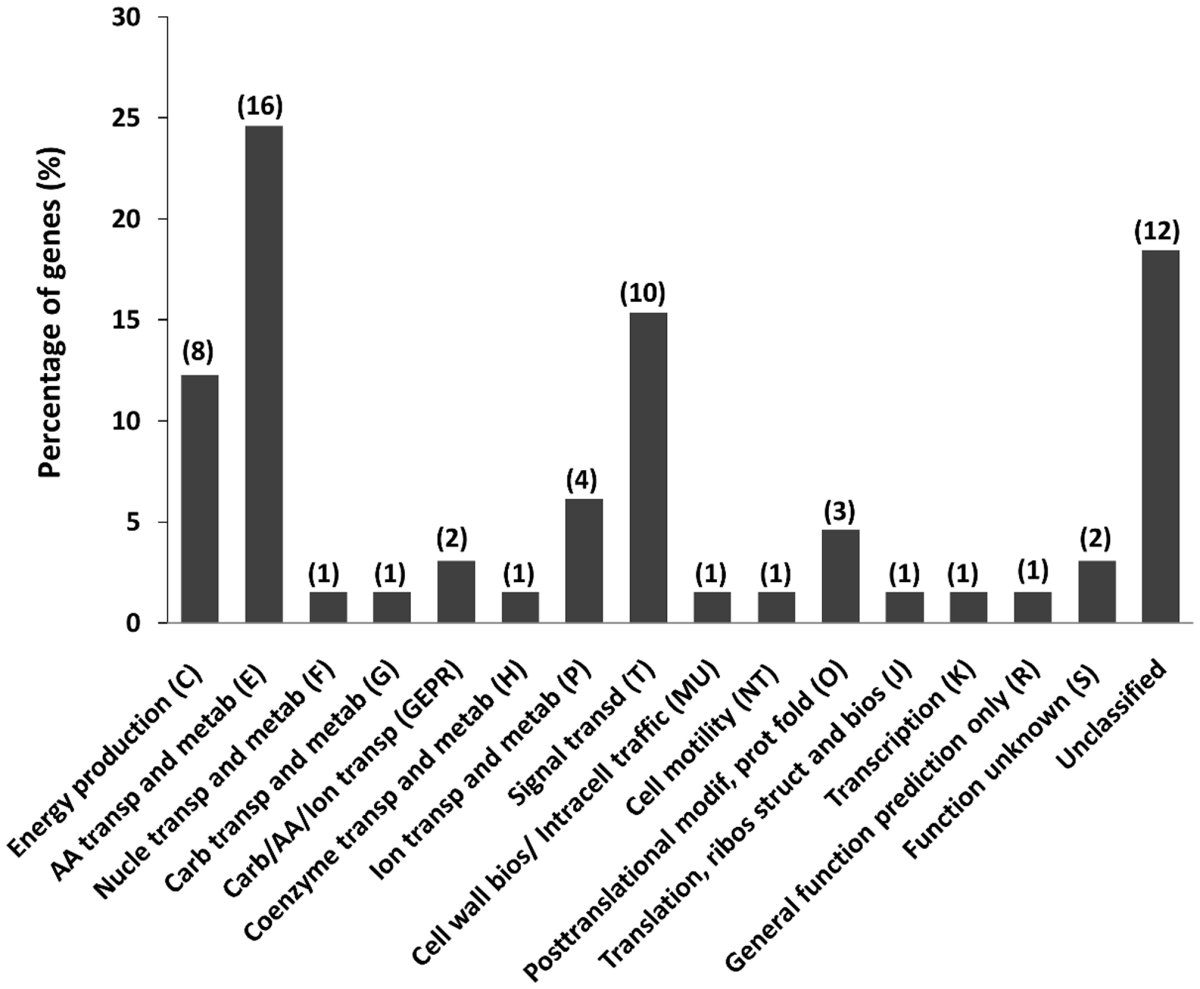
Distribution of the differentially expressed genes according to the clusters of orthologous groups of proteins (COG) classification (in percentage). The numbers in parentheses indicate the numbers of differentially expressed genes for each COG.

The distribution of the differentially expressed genes according to the COGs database is shown in Figure 4. A large portion (18.5%) of the differentially expressed genes did not belong to any COG class. The genes belonging to the amino acid transport and metabolism (E), signal transduction mechanisms (T), and energy production and conversion (C) classes were the most abundant (24.6%, 15.4%, and 12.3%, respectively). Because these three categories represent only 8.3%, 9.4%, and 6.8% of the predicted CDSs, respectively, in the whole *D. hydrothermalis* genome (MicroScope accession DESAMv2_DESAMv2), these categories are likely to be important for pressure adaptation.

Fifteen genes were differentially expressed in the 10 *vs.* 0.1 MPa and 26 *vs.* 0.1 MPa conditions but not in the 26 *vs.* 10 MPa condition (Table 1). All were down-regulated at high pressure compared to atmospheric pressure. Among these, three genes encoded heat shock proteins Hsp20 (DESAMv2_20304, DESAMv2_20661–20662). This expression pattern is in agreement with the optimal growth hydrostatic pressure (26 MPa) of the strain. Induction of stress proteins encoding genes as a function of hydrostatic pressure has been also reported in *Escherichia coli* [20] and in the piezophile *Photobacterium profundum* [21]. Three genes were involved in anthranilate metabolism and were linked to glutamate biosynthesis (DESAMv2_21553- DESAMv2_21555). Five genes belonged to the signal transduction mechanisms category (DESAMv2_21455-DESAMv2_21459) and could be involved in regulating the response to hydrostatic pressure variations. These 15 genes are thus involved in the adaptation to hydrostatic pressures above 0.1 MPa. The absence of significant variations in the 26 *vs.* 10 MPa condition could be explained by the fact that the response mechanisms were already active at 10 MPa.

Thirty-two genes were differentially expressed when the hydrostatic pressure was higher than 10 MPa (Table 1). These included genes linked to aromatic amino acid and glutamate metabolism, which were down-regulated, and genes involved in energy metabolism. The gene cluster DESAMv2_21433-DESAMv2_21438, which encodes the transmembrane Hmc complex [22], was up-regulated at pressures above 10 MPa. These data suggest that these mechanisms are engaged at the highest pressures.

A final group corresponded to 12 genes that were differentially expressed only at 10 *vs.* 0.1 MPa. The adaptation mechanisms in which these genes may be involved would only occur at hydrostatic pressures below HP_t_. This group included a gene encoding for an alcohol dehydrogenase (DESAMv2_20447), which was found to play an important role in *Desulfovibrio vulgaris* Hildenborough energy metabolism [23], as well as genes involved in iron transport (DESAMv2_20613-DESAMv2_20614, DESAMv2_21115) and cobalt binding (DESAMv2_21928).

### Aromatic amino acid and glutamate biosynthesis

As mentioned above, the largest number of differentially expressed genes belonged to the amino acid transport and metabolism category (Table 1). These genes were linked to the biosynthesis of glutamate and aromatic amino acids. DESAMv2_21946 encoded a glutamine synthetase, which was down-regulated when the pressure was higher than 10 MPa, together with a nitrogen regulatory protein PII (DESAMv2_21452), which modulated the activity of the former [24]. The gene cluster DESAMv2_21549–21557 and the DESAMv2_21414::trpB gene (encoding a tryptophan synthase subunit) are involved in the shikimate and aromatic amino acid biosynthetic pathways (Figure S1). Shikimate and chorismate are precursors in the biosynthesis of aromatic amino acids (tryptophan, phenylalanine, and tyrosine). The shikimate pathway is linked to glutamate metabolism through one of the key enzymes of the tryptophan biosynthesis pathway, anthranilate synthase (DESAMv2_21553–21554), which requires glutamine for its activity. The observed down-regulation of gene expression at high pressure would decrease the utilization of glutamine and reduce glutamate turnover, which could in turn lead to glutamate accumulation in cells. Considered together, these data suggested that glutamate accumulates in cells at high hydrostatic pressure. It is noteworthy that three genes encoding tryptophan biosynthetic enzymes (anthranilate synthase (DESAMv2_21553–21554) and anthranilate phosphoribosyltransferase (DESAMv2_21555)) were down-regulated at 10 *vs.* 0.1 MPa, suggesting their specific involvement in the 0.1-to-10 MPa pressure adaptation range, defined as the first-level response to hydrostatic pressure increase.

**Figure 5:**
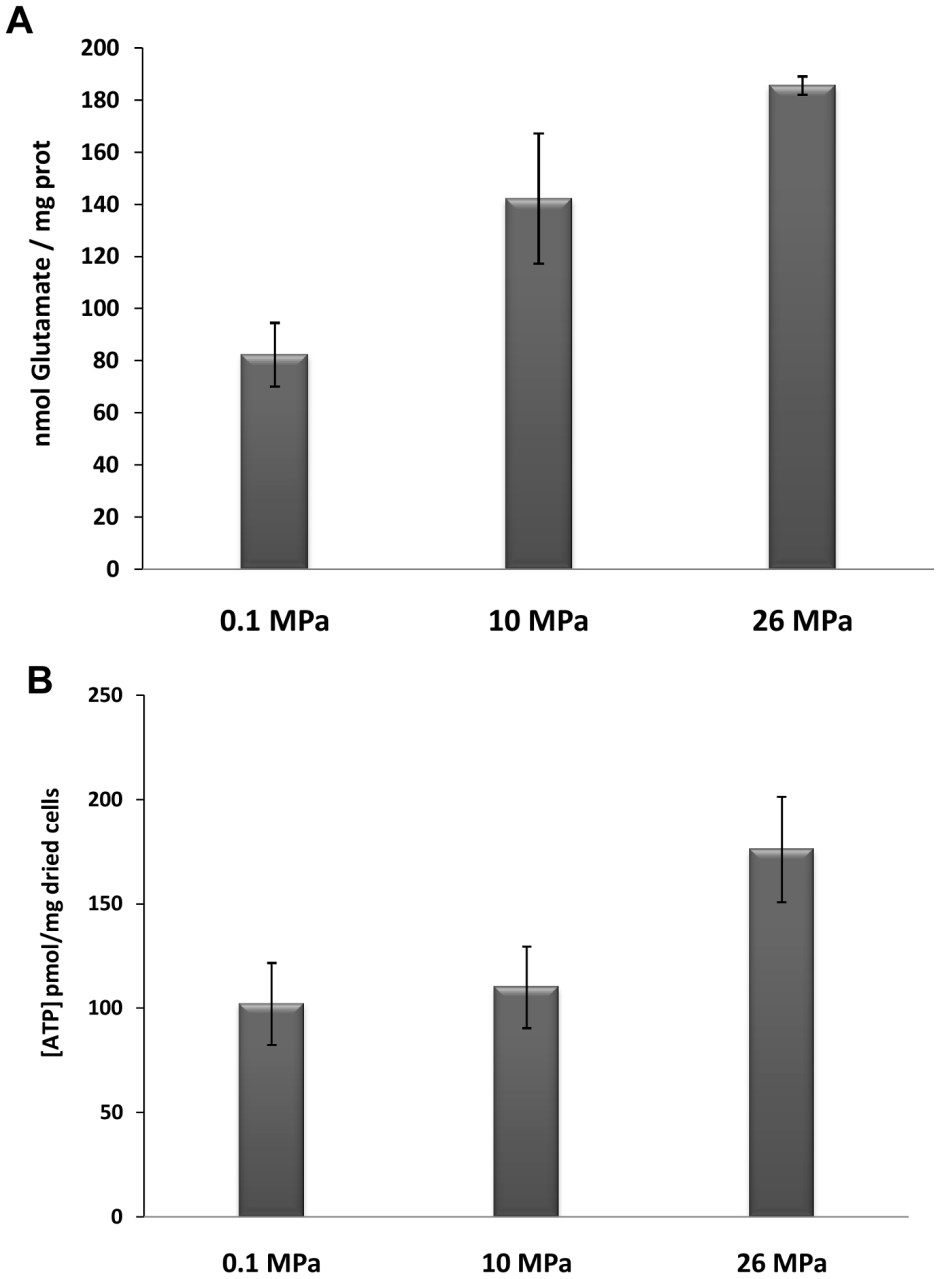
Quantitation of glutamate levels (A) and intracellular ATP (B) in *D. hydrothermalis* cells grown under different pressure conditions.

Because our data suggested glutamate accumulation in *D. hydrothermalis* at high pressure, we measured the amount of glutamate in cells. The concentration of intracellular glutamate in cells collected during the exponential phase is shown in Figure 5A. The intracellular glutamate concentration was 82.3 (±12.2) nmol/mg prot in cells grown at atmospheric pressure, whereas a concentration of 185.6 (±3.5) nmol/mg prot was observed in cells grown at 26 MPa, corresponding to a 2.25-fold increase. An intermediate concentration (142.2 (±24.9) nmol/mg prot) was observed when cells were cultured at 10 MPa. These data show that glutamate accumulates within cells at high hydrostatic pressures and that glutamate accumulation increases with increasing hydrostatic pressure.

### Energy metabolism regulation

Among the differentially expressed genes belonging to the energy metabolism category (Table 1), six genes encoded for the transmembrane electron transport complex Hmc (DESAMv2_21433-DESAMv2_21438). The Hmc complex has been extensively studied in *Desulfovibrio* species and has been found to be involved in the electronic link between periplasmic hydrogen oxidation and cytoplasmic sulfate reduction [22]. The Hmc complex, composed of six subunits (HmcA-F) located at the inner membrane, is encoded by a multicistronic unit called the *hmc* operon [25]. In *D. hydrothermalis*, the *hmc* genes were up-regulated when cells were cultured at 26 MPa. Surprisingly, the RNA-seq analysis showed that two genes of the *hmc* operon are down-regulated when cells were cultured at 10 MPa, compared with atmospheric pressure. Moreover, the gene encoding a Fe-containing alcohol dehydrogenase (DESAMv2_20447) was down-regulated at 10 MPa *vs.* 0.1 MPa. It has been proposed that this enzyme contributes to energy metabolism in *D. vulgaris* Hildenborough through the alcohol-to-proton-gradient pathway [23]. Thus, two distinct response patterns appear to occur when the hydrostatic pressure is below or above the HP_t_ threshold.

To determine whether the hydrostatic pressure-induced modifications to energy metabolism indicated by RNA-seq affected cellular ADP and ATP pools, these compounds were quantified in cells grown at each of the three different pressures. The ATP concentrations in cells grown at 0.1, 10, and 26 MPa are presented in Figure 5B. ATP levels varied from 102 (±19.6) pmol/mg dried cells to 176 (±25.3) pmol/mg dried cells, with the highest levels observed in cells grown at the *in situ* hydrostatic pressure (26 MPa). The measured ADP/ATP ratio was 0.10 (±0.08) in cells grown at 26 MPa and 0.22 (±0.11) and 0.44 (±0.13) when cells were cultured at 10 MPa and 0.1 MPa, respectively. These data show that the phosphorylation process is more efficient under *in situ* hydrostatic pressure conditions (26 MPa) than at lower pressures.

## Discussion

Genetic and biochemical experiments performed on deep-sea microorganisms have revealed that both physiological and structural adaptations are essential for high-pressure life [3]. Bacteria engage in a global response to hydrostatic pressure, involving not only the derepression of functions to facilitate cellular adaptation but also the enhanced activity of enzymes and regulatory proteins. It has also been suggested that the composition of membrane lipids, the structure and abundance of proteins, and the accumulation of solutes (piezolytes) could influence bacterial growth in deep-sea environments [20], [26], [27]. Microarray experiments on *Photobacterium profundum* led to the identification of 260 genes that were differentially expressed under different hydrostatic pressure growth conditions, most of which encoded proteins involved in amino acid and ion transport, protein folding, and glycolysis [9].

Our work represents the first transcriptome-level analysis of the effect of hydrostatic pressure on a piezophilic sulfate-reducing bacterium, *Desulfovibrio hydrothermalis*. This bacterium originates from a deep-sea hydrothermal vent on the East-Pacific Rise at a depth of 2,600 m, which corresponds to an *in situ* hydrostatic pressure of 26 MPa. Only 65 genes were found to be differentially expressed depending on the hydrostatic pressure. These genes are distributed into four main categories: aromatic amino acid and glutamate metabolism, energy metabolism, signal transduction, and unknown function. Notably, the majority of these genes are located in close proximity to one another, within three main gene clusters. The first cluster includes 10 genes (DESAMv2_21548–21557) that are mainly involved in aromatic amino acid metabolism. The second cluster is composed of 9 genes (DESAMv2_21431–21439) that mainly encode the Hmc complex, which is involved in energy metabolism. The last cluster includes 9 genes (DESAMv2_21453–21461) that mainly encode regulators and proteins of unknown function. These latter proteins could specifically function in the adaptation to hydrostatic pressure, opening the way to functional genomic investigations.

Because the response of *D. hydrothermalis* to hydrostatic pressure at the transcriptomic level involves relatively few genes and clusters, we can assume that its adaptation to hydrostatic pressure is quite specific and involves only a limited number of mechanisms, even if we can not exclude the additional involvement of posttranscriptional processes. One of these mechanisms is the accumulation of glutamate at high hydrostatic pressure. Analysis of the metabolic pathways affected by pressure suggests that this accumulation is driven by reductions in both glutamine synthase activity and aromatic amino acid biosynthesis. In *P. profundum* SS9, several genes involved in glutamate metabolism have also been shown to be differentially expressed with pressure [9], [21], [28]. Ikegami *et al*. [29] have shown that the expression of the glutamine synthase gene *glnA* of *Shewanella violacea* is positively regulated by hydrostatic pressure. In addition, when *Methanocaldococcus jannaschii* was shocked from 0.8 to 50 MPa over 15 min, a glutamine amidotransferase-encoding gene was down-regulated [10]. These findings suggest that glutamate/glutamine metabolism is of great importance for adaptation to hydrostatic pressure in a highly metabolically diverse set of deep-sea micro-organisms. Because increased hydrostatic pressure can alter the conformation, packing, and intermolecular interactions of macromolecules, cells may offset these effects through the accumulation of protein-stabilizing solutes [1]. In the case of hydrostatic-pressure-change conditions, the *de novo* biosynthesis of intracellular molecules could be activated not only to compensate for exchanges in the extracellular environment but also to accumulate stabilizing molecules. The deep-sea bacterium *P. profundum* strain SS9 was found to accumulate β-hydroxybutyrate at high hydrostatic pressure [26]. The data presented here show that *D. hydrothermalis* accumulates glutamate at high hydrostatic pressure, highlighting the role of glutamate as a major piezolyte in the adaptation of this *Desulfovibrio* sp. to hydrostatic pressure.

This work also highlights that the expression of genes related to energy metabolism is affected by hydrostatic pressure. This was also shown in the piezophiles *S. violacea* and *P. profundum*, where terminal cytochrome c oxidase and quinol oxidase have been found to be differentially expressed depending on the hydrostatic pressure [11], [30]. ATP levels may fluctuate significantly and reversibly with metabolic stress [31]. The ATP level in *D. hydrothermalis* cells grown at 26 MPa was higher than in cells grown at either 0.1 MPa or 10 MPa. Similarly, the ADP/ATP ratio shows that ATP regeneration is more efficient at high hydrostatic pressure (26 MPa) than at lower hydrostatic pressures (0.1 MPa and 10 MPa). These results suggest that *D. hydrothermalis* modifies its energy metabolism depending on the pressure growth conditions. Several mechanisms for generating the ATP required for the growth and maintenance of *Desulfovibrio* spp. have been proposed, including substrate-level phosphorylation and oxidative phosphorylation [32]. Sulfate reduction is a respiratory process that leads to oxidative phosphorylation through an electron transfer pathway [33]. This electron transport chain involves cytoplasmic dehydrogenases and terminal reductases, as well as transmembrane electron transport complexes, one of which is the Hmc complex [34]. The more efficient energy metabolism of *D. hydrothermalis* at 26 MPa than at lower hydrostatic pressures could be linked to a larger abundance of the Hmc complex at 26 MPa, which is involved in the oxidative phosphorylation process.

The gene expression patterns observed from the transcriptome analyses reveal three groups of genes. Expression of the first group is regulated only when the hydrostatic pressure does not exceed 10 MPa. Expression of the second group is regulated whenever the hydrostatic pressure exceeds 0.1 MPa, and the last is regulated only when the hydrostatic pressure exceeds 10 MPa. These patterns suggest that *D. hydrothermalis* uses at least three different adaptation mechanisms, according to a hydrostatic pressure threshold (HP_t_) that is estimated to be above 10 MPa. The following scenario can be envisioned: (*i*) when the increase in pressure is below HP_t_, adaptation mainly involves the modification of energy metabolism through the alcohol-to-proton-gradient pathway [23] and the Fe^2+^ transport systems; (*ii*) the second mechanism, activated as soon as the hydrostatic pressure increases, mainly involves modifications of tryptophan metabolism, which in turn could influence on intracellular glutamate level, and genes encoding regulators and proteins of unknown function; (*iii*) the last mechanism, which is activated only at high hydrostatic pressure (above HP_t_), mainly involves directly glutamate metabolism and energy metabolism.

The gene expression data obtained from this study provide a valuable resource for further functional studies of *Desulfovibrio* spp. pressure-adaptation mechanisms and provide insights into the underlying molecular systems in sulfate-reducing bacteria.

## Supporting Information

Figure S1 **KEGG map of phenylalanine, tyrosine, and tryptophan biosynthesis.**
(PDF)

Table S1 **Analysis of RNA-seq data mapped to the** ***D. Hydrothermalis*** **genome.**
(PDF)
