# Supplementary material for: Transcriptomics Reveal Several Gene Expression Patterns in the Piezophile Desulfovibrio hydrothermalis in Response to Hydrostatic Pressure
Source: PLoS One. 2014 Sep 12;9(9):e106831. doi: 10.1371/journal.pone.0106831 (PMC4162548; doi:10.1371/journal.pone.0106831)
Supplement: Table S1 — Analysis of RNA-seq data mapped to the D. Hydrothermalis genome. (PDF) [file pone.0106831.s002.pdf]

Table S1. Analysis of RNA-seq data mapped to the *D. hydrothermalis* genome.

	Replicate 1			Replicate 2		
	0.1 MPa	10 MPa	26 MPa	0.1 MPa	10 MPa	26 MPa
Total number of reads	64 743 038	57 771 788	54 984 766	50 667 790	48 797 314	46 986 944
Reads mapped to genome	49 825 927	41 935 232	40 730 491	42 077 649	39 312 831	37 888 861
% total mapped	76.96	72.59	74.08	83.05	80.56	80.64
Reads properly paired	22 572 854	14 780 526	15 253 376	27 673 006	22 214 646	22 699 170
% properly paired	34.87	25.58	27.74	54.62	45.52	48.31
