# Supplementary material for: Transcriptomics Reveal Several Gene Expression Patterns in the Piezophile Desulfovibrio hydrothermalis in Response to Hydrostatic Pressure
Source: PLoS One. 2014 Sep 12;9(9):e106831. doi: 10.1371/journal.pone.0106831 (PMC4162548; doi:10.1371/journal.pone.0106831)

Figure S1. KEGG map of phenylalanine, tyrosine, and tryptophan biosynthesis. The Enzyme Commission numbers identified in the *D. hydrothermalis* genome are highlighted in green and yellow. The genes down-regulated at 26 MPa *vs.* 0.1 MPa are highlighted in yellow. Tag loci of the corresponding enzyme are surrounded in yellow.

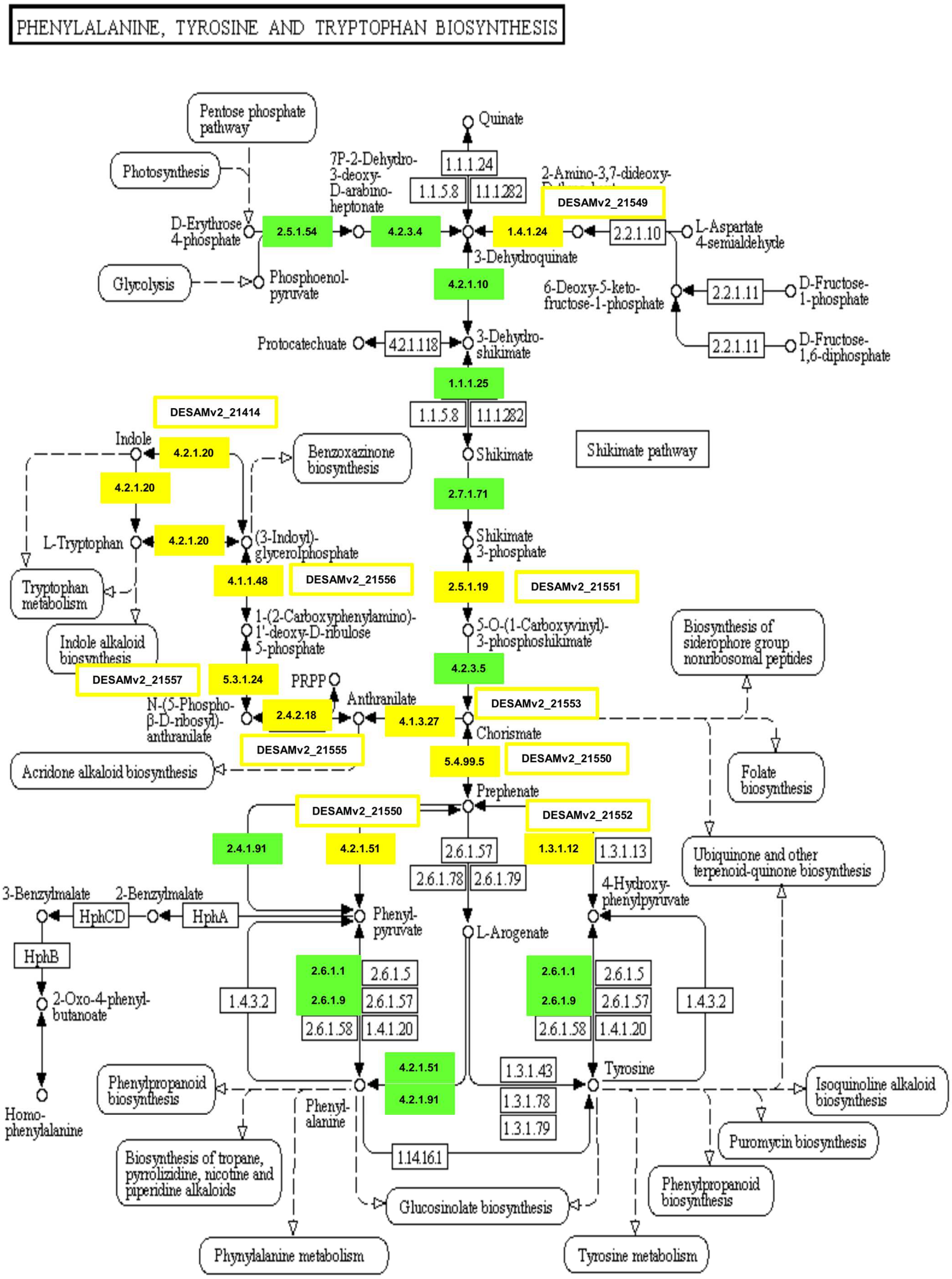

Supplement: Figure S1 — KEGG map of phenylalanine, tyrosine, and tryptophan biosynthesis. (PDF) [file pone.0106831.s001.pdf]
